# Brevities

**Published:** 1868-06

**Authors:** 


### BREVITIES.

The annual meeting of the Northern Ohio Dental Association was held in Cleveland a few days ago. There was a very good representation of its membership present, and in addition, some visitors. It was a very interesting meeting; the action of which, in some particulars, at least, will make impressions that will not soon be forgotten, especially by those more particularly interested. The discussion upon principles and practice was very good, indeed.

This is one of the old societies, and has the reputation of being an active working society—doing much good for the profession within the sphere of its influence. We think this meeting fully sustained its reputation.

---

Editors Register :—Within the past four days my attention has been called to specimens of artificial teeth manufactured by C. H. Eccleston, of Oxford, N. Y.; and feeling it a duty to encourage every scientific advance in our profession, I would say, that in my opinion they are superior in natural form and appearance to any that I have seen.

Instead of the flat, formal appearance of most of the teeth sent us, the gums are beautifully carved to resemble the irregularity of the natural gums. The canines and gums above them are brought out as they should be, more prominent than the laterals, giving a better shape to the whole denture than we have been able to give with any other teeth yet brought to our notice. The gums are very life-like, and taken as a whole they come the nearest to what we all so much admire (continuous gum), of any I have seen.

The manufacturer assures us that he has a great variety of patterns, and ready to add more as the profession may suggest, and is willing to put them in comparison as to strength with any other teeth manufactured.

And what is more, he can afford and will sell them to us at about one-half that we are now paying for our best teeth of other makers. Let us encourage merit, by giving them a fair trial; and if they prove to be what he promises, we shall gain two points, a beautiful tooth at a reasonable price.

Progress.

